# Machine Learning to Predict Drug-Induced Liver Injury and Its Validation on Failed Drug Candidates in Development †

**DOI:** 10.3390/toxics12060385

**Published:** 2024-05-24

**Authors:** Fahad Mostafa, Victoria Howle, Minjun Chen

**Affiliations:** 1 Department of Mathematics and Statistics, Texas Tech University, Lubbock, TX 79409, USA; fahad.mostafa@ttu.edu (F.M.); victoria.howle@ttu.edu (V.H.); 2 Division of Bioinformatics and Biostatistics, the US FDA’s National Center for Toxicological Research, Jefferson, AR 72029, USA

**Keywords:** machine learning, drug-induced live injury, liver toxicity, random forest, multilayer perceptron, failed drug candidates

## Abstract

Drug-induced liver injury (DILI) poses a significant challenge for the pharmaceutical industry and regulatory bodies. Despite extensive toxicological research aimed at mitigating DILI risk, the effectiveness of these techniques in predicting DILI in humans remains limited. Consequently, researchers have explored novel approaches and procedures to enhance the accuracy of DILI risk prediction for drug candidates under development. In this study, we leveraged a large human dataset to develop machine learning models for assessing DILI risk. The performance of these prediction models was rigorously evaluated using a 10-fold cross-validation approach and an external test set. Notably, the random forest (RF) and multilayer perceptron (MLP) models emerged as the most effective in predicting DILI. During cross-validation, RF achieved an average prediction accuracy of 0.631, while MLP achieved the highest Matthews Correlation Coefficient (MCC) of 0.245. To validate the models externally, we applied them to a set of drug candidates that had failed in clinical development due to hepatotoxicity. Both RF and MLP accurately predicted the toxic drug candidates in this external validation. Our findings suggest that in silico machine learning approaches hold promise for identifying DILI liabilities associated with drug candidates during development.

## 1. Introduction

Drug-induced liver injury (DILI) continues to be a significant challenge for the pharmaceutical industry and regulatory organizations [1]. DILI is a complex safety issue characterized by various underlying processes, varying degrees of severity, population-specific differences, and the inherent challenges in categorizing the risk connected to medications, especially those recently granted marketing approval [2,3,4]. To prevent drugs with DILI liability from entering the market, it is crucial to enhance the ability to predict the potential risk of DILI in humans before obtaining marketing approval [5]. This improvement would lead to safer pharmaceuticals and more cost-effective drug development. In addition, prompt discontinuation of the development of drug candidates with a high propensity to cause DILI can result in the timely use of resources [6].

In silico models have gained popularity among researchers due to their rapid development times, cost-effectiveness and no requirement of physical substances, although experimental approaches such as in vitro and in vivo assays are still integral in the assessment of DILI risk [5,7]. Typically, in silico DILI prediction models have been developed and validated using marketed drugs, neglecting testing on drug candidates in the developmental stages [8]. Recognizing the challenge of the “translational gap” between drug development and clinical research [9,10], it becomes imperative to evaluate the performance of these in silico models using drug candidates that have failed during the drug development process.

In this study, we employed a large DILI likelihood dataset [11] created by the experts from the NIH LiverTox consortium [12]. We conducted a comparative analysis of various machine learning and deep learning algorithms to develop Quantitative Structure–Activity Relationship (QSAR) models, utilizing in-house Mold2 chemical descriptors. The evaluation through 10-fold cross-validation was used to select the model with the highest performance in terms of accuracy (ACC) or Matthews Correlation Coefficient (MCC). Subsequently, these models underwent external validation using a set of drug candidates that failed in clinical development due to hepatotoxicity in humans.

## 2. Materials and Methods

### 2.1. Data and Annotation for DILI Likelihood

Medications can be classified in terms of their likelihood to cause liver injury in humans. The NIH LiverTox consortium has created a five-point classification for measuring the possibility that a medicine caused DILI. This classification is mostly based on the published literature. A drug is categorized by likelihood score on DILI [11] as category A (well known for DILI), Category B (highly likely to cause DILI), Category C (probably linked to DILI), Category D (possible cases for DILI), Category E (no evidence for DILI), or Category $E^{*}$ (suspected but no convincing DILI cases).

In this study, the dataset is reclassified into drugs with the likelihood to cause DILI (DILI group) and without the likelihood to cause DILI (non-DILI group). The likelihood of YES to DILI is enumerated as 1 for categories A, B, and C, and NO to DILI as 0 with E and E*. The training set comprises 240 DILI-positive, which includes 61 drugs belonging to group A, 80 of group B, and 99 of group C, and 335 DILI-negative with 206 of group E and 129 of group E*. Notably, the DILI-positives contained 34 medications with Black Box Warnings or withdrawn from the market due to DILI concern. An independent dataset were used as the external validation, including 22 drug candidates that were terminated in development due to liver toxicity [13]. 

### 2.2. Molecular Descriptors

Here, we used Mold2 to calculate 777 1D, and 2D molecular descriptors to capture critical two-dimensional chemical structural information associated with drugs. Mold2 is a software package developed by the FDA NCTR for fast calculating a large and diverse set of molecular descriptors encoding two-dimensional chemical structure information [14]. Compared with those calculated from commercial software on published datasets Mold2 descriptors provides similar or better performance to represent sufficient structural information [14]. 

### 2.3. Model Development

In the binary classification of machine learning, the data are represented as a set of pairs (x,y), where x denotes the input features and y represents the binary target variable. Mathematically, the input features are typically denoted as a real-valued vector x∈ $R^{n}$, where n is the dimensionality of the feature space. The target variable y is a binary label, taking on one of two values: 0 or 1. The goal of binary classification is to learn mapping from input features to the binary labels, which is represented as a function $f:R^{n}\to \{0,1\}$. This mapping is learned through a training process using a labeled dataset, and the objective is to find a decision boundary or a hypothesis h(x) that accurately predicts the binary label y based on the input features x in a way that minimizes a suitable loss or cost function. The framework of model development for DILI prediction includes the following:

- Feature selection: Not all features may be relevant for predicting DILI. Feature selection techniques can help identify the features that have the most impact on the prediction.
- Model selection: There are various machine learning algorithms that can be used, such as decision trees (DTs), RFs, support vector machines (SVMs), naive Bayes classifier, and neural networks. The choice of the model depends on the characteristics of the data and the specific problem at hand.
- Model training and hyperparameter tuning: The dataset is divided into training and validation sets. The model is developed on the training set using the selected algorithm and the chosen performance metric. Hyperparameters of the machine learning model can significantly affect its performance. Techniques like grid search or random search are used to find the optimal hyperparameters.
- Model evaluation: The model’s performance is assessed on the validation set using appropriate evaluation metrics as detailed below.
- Model test and interpretation: After finding the best model with optimal hyperparameters, it will be tested on an independent test set to get an estimate of its real-world performance. Additionally, interpretability techniques can help understand which features are driving the predictions and provide insights into the underlying mechanisms of DILI.

#### 2.3.1. Feature Selection from Chemical Descriptors

**Figure 1:**
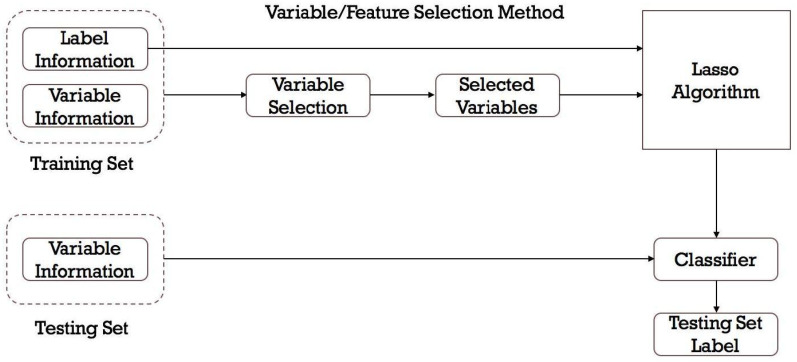
Feature selection for DILI prediction.

Feature selection helps identifying the features that have the most impact on the prediction. It combats computational complexity, overfitting, and improves model interpretability by selecting the most relevant characteristics. Choosing influential features helps a model generalize to new data, minimizes noise, and adds to more efficient model training (Figure 1). 

To assess the importance of input features, we analyzed the coefficients (weights) associated with each feature in the logistic regression model [15]. The absolute values of these coefficients represent the feature’s importance. The larger the absolute coefficient, the more important the feature is in predicting the binary outcome. 

The logistic regression model represents the relationship between the features and the binary outcome (0 or 1) using the logistic function (sigmoid function). The model is defined as follows:

$$\begin{array}{cc} X: & \mathrm{Feature}\;\mathrm{matrix}\;\mathrm{with}\;\mathrm{dimensions}\;\left(m\times n\right)\\ Y: & \mathrm{Binary}\;\mathrm{target}\;\mathrm{variable}\;\left(0\;\mathrm{or}\;1\right)\;\mathrm{with}\;\mathrm{dimensions}\;\left(m\times 1\right)\\ \theta : & \mathrm{Vector}\;\mathrm{of}\;\mathrm{parameters}\;(\mathrm{weights})\;\mathrm{with}\;\mathrm{dimensions}\;\left(n\times 1\right)\\ Z: & \mathrm{Linear}\;\mathrm{combination}\;\mathrm{of}\;\mathrm{features}\;\mathrm{and}\;\mathrm{parameters}:\;Z=X\cdot \theta \\ h\left(\theta \right): & \mathrm{Logistic}\;(\mathrm{sigmoid})\;\mathrm{function}:\;h\left(\theta \right)=\frac{1}{1+e^{-Z}} \end{array}$$

The logistic regression model can then be defined as

$$P(Y=1|X)=h(\theta )=\frac{1}{1+e^{-X\theta }}$$

To reduce overfitting, Lasso regularization is adopted during logistic regression, which encourages sparse coefficient values, making feature selection more explicit [16]. The logistic regression model with L1 regularization is represented as follows with the defined objective function:

$$J(\theta )=-\frac{1}{m}\sum_{i=1}^{m}\left[y^{\left(i\right)}log\left(h_{\theta }\left(x^{\left(i\right)}\right)\right)+\left(1-y^{\left(i\right)}\right)log\left(1-h_{\theta }\left(x^{\left(i\right)}\right)\right)\right]+\lambda \sum_{j=1}^{n}\left|\theta_{j}\right|$$

Here, J(θ) is the logistic regression cost function with Lasso regularization, m is the number of training samples, n is the number of features, θ represents the model parameters (weights), $x^{\left(i\right)}$ is the feature vector of the i-th sample, and $h_{\theta }\left(x^{\left(i\right)}\right)$ is the logistic function. The λ term controls the strength of Lasso regularization. The Lasso regularization term encourages some of the θ coefficients to become exactly zero, which effectively selects a subset of features. The features with non-zero coefficients in the trained model are considered important for the classification task. 

#### 2.3.2. Model Selection and Mathematical Analysis

Model selection is an important component of developing predictive models since algorithms have different capabilities for modeling. In our current DILI study, binary classification was considered. 

If x is the input features of dimension n, then y is the binary class with class labels 0 and 1. If θ is the model parameters and $H_{\theta }$ is the predicted P(y=1∣x,θ), the SVM classifier’s prediction probability is:

$$H_{\theta }(x)=sign\left(\theta^{T}x+b\right),$$

$H_{\theta }(x)$ predicts the class label, θ is the weight of the parameters and b is the bias. The objective function to minimize is

$$\underset{\theta ,b}{min}\frac{1}{2}∥\theta {∥}^{2}+C\sum_{i=1}^{m}max\left(0,1-y^{\left(i\right)}\left(\theta^{T}x+b\right)\right)$$

In the case of the DT classifier [17], it recursively partitions the feature space into regions by selecting split based on Gini impurity or the entropy index. Random forest (RF) is an ensemble of DTs [18,19]. Each tree is trained on a bootstrap sample, and the final prediction is obtained by aggregating predictions of individual trees. A deep learning-based classifier, which is known as multilayer perceptron (MLP), is also used to train the model. The mathematical representation of a neural network is as follows:

$$H_{\theta }(x)=softmax\left(W^{\left(2\right)}\cdot ReLU\left(W^{\left(1\right)}\cdot x+b^{\left(1\right)}\right)+b^{\left(2\right)}\right)$$

where $W^{\left(1\right)}$ and $W^{\left(2\right)}$ are weight matrices, and $b^{\left(1\right)}$ and $b^{\left(2\right)}$ are the biases. The cross-entropy loss function is

$$J(W,b)=-\frac{1}{m}\sum_{i=1}^{m}\sum_{j=1}^{K}\left[y_{ij}log\left(H_{ij}\right)\right]$$

The KNN classifier assigns a class label C to a new instance $x_{new}$ based on the majority class among its k-nearest neighbors as follows:

$$C\left(x_{new}\right)={argmax}_{c}\sum_{i=1}^{k}\delta \left(y_{i},c\right)$$

where $C\left(x_{new}\right)$ is the predicted class label for the $x_{new},c$ iterates the overall possible class labels, and $\delta \left(y_{i},c\right)$ is the Kronecker delta, which is 1 if $y_{i}=c$ and 0 otherwise. To find the KNN of $x_{new}$, the Euclidean distance metric is used as $d\left(x_{i},x_{new}\right)=$ $\sqrt{\sum_{j=1}^{m}{\left(x_{ij}-x_{new,j}\right)}^{2}}$, where m is the number of features in the data. Stochastic Gradient Descent (SGD) and Adaptive Moment Estimation (ADAM) are two optimization methods that are often used in machine learning models to minimize loss functions. SGD iteratively updates model parameters in logistic regression by examining small portions of training data, improving convergence speed and scalability [20]. These techniques let SVM discover the best hyperplane by repeatedly modifying the separation margins [21]. ADAM aids training in MLP by fine-tuning the network’s weights and biases, allowing it to approximate complicated correlations between inputs and outputs [22].

#### 2.3.3. Model Evaluations and Statistical Analysis

To determine performance measures, predictions from each of the 100 iterations of the 10-fold cross-validation were compared with their actual DILI risk classifications. Each 10-fold cross-validation for the 2-class DILI prediction models produced a 2-by-2 confusion matrix.

A panel of statistical matrices are used for investigating machine learning models [23], such as accuracy (*ACC*), precision, recall, F1 score (*F*1), and *MCC*. The predictions made by a classifier can be categorized as true positive (*TP*), true negative (*TN*), false positive (*FP*), and false negative (*FN*).

$$ACC=\frac{TP+TN}{TP+TN+FP+FN}$$

$$Precision=\frac{TP}{TP+FP}$$

$$Recall=\frac{TP}{TP+FN}$$

$$F1=\frac{2\cdot Precision\cdot Recall}{Precision+Recall}$$

$$MCC=\frac{TP\cdot TN-FP\cdot FN}{\sqrt{\left(TP+FP)(TP+FN)(TN+FP)(TN+FN\right)}}$$

#### 2.3.4. Applicability Domain Analysis

Determining the applicability domain [24,25] often involves using various statistical and cheminformatics techniques, such as clustering analysis, distance measures, or domain-specific rules. These methods help assess whether new data points or compounds fall within the established domain. Knowing the domain of application enables users to apply the model to data that are comparable to the training data, enhancing trust in their robustness and generalizability. Normally, a hypothesis is considered for finding optimal prediction space in the 2D visualization of the prediction data points.

**Hypothesis:**  The optimal prediction space (OPS) from machine learning models implements a variation of principal component analysis (PCA). In the PCA method, the data are centered on the mean of each parameter range $\frac{x_{max}-x_{min}}{2}$ rather than the standardized mean value. As a result, it creates a new orthogonal coordinate system called the OPS coordinate system. The minimum and maximum values of the produced data points on each axis of the OPS coordinate system form the OPS border.

Here, the RF classifier and the Mahalanobis distance are utilized to apply the applicability domain [26] for evaluating the eligibility of new data points for prediction. Given a training dataset consisting of input features denoted as $X_{train}$ and corresponding class labels $y_{train}$, and a trained classifier model RF, the Mahalanobis distance, $DO\left(x_{new}\right)$, is calculated for a new data point $x_{new}$ using the mean and covariance matrix of $X_{train}$. A threshold value, T, determined by a chosen significance level α and degrees of freedom, is utilized. The RF classifier predicts the class label $y_{pred}$ for $x_{new}$. If $DO\left(x_{new}\right)$ is less than $T,x_{new}$ is within the applicability domain; otherwise, it’s outside. The Mahalanobis distance’s comparison with T dictates the data point’s classification. 

## 3. Results

### 3.1. Assessments of Feature Selection

**Figure 2:**
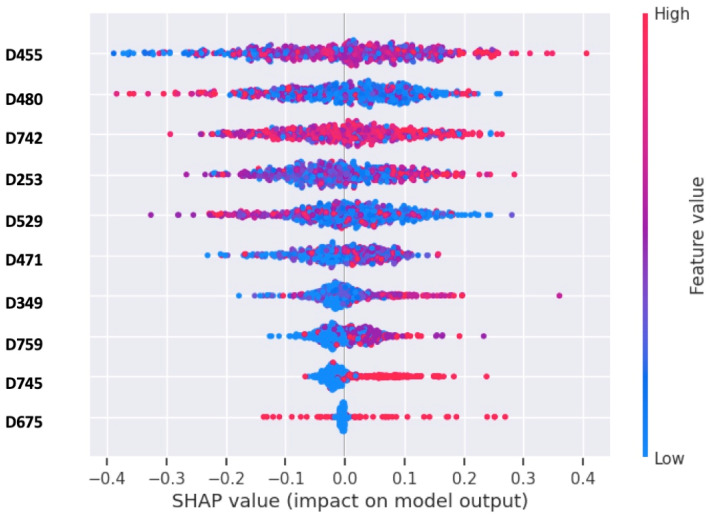
Shapley values used to explain the prediction of a model by attributing the contribution of 10 most important features out of 168 top features to the prediction.

Feature selection is critical for machine learning to solve the issues associated with high-dimensional DILI datasets. Here, we used a regularized logistic regression method for identifying the most important features. The dataset was split into training and validation sets with an 80–20 ratio by using a simple random sampling technique. Then, we selected the top important features for classification based on their root mean squared error (RMSE) and λ using least absolute shrinkage and selection operator (lasso) and transformed the training and testing sets to include only the selected features. For doing this we selected a range of values for the regularization parameter λ using cross-validation. For each value of λ, we fit a lasso regression model on the training data. Then, we used a validation dataset to evaluate the performance of each lasso model. Once the optimal λ was determined, we fit the lasso regression model with this λ value on the entire dataset to obtain the final model. Using lasso with 10-fold CV, 168 top-ranked molecular descriptors were selected with the best λ = 0.01 and minimum RMSE of 0.466. The model employed feature selection using L1 regularization with logistic regression and calculated *p*-values for the selected features. After standardizing the features, it fit the lasso model to the training data, identifying the features with non-zero coefficients and the lowest *p*-values. From the 168 top-ranked descriptors obtained through the lasso variable selection strategy, we extracted the 10 most valuable features with the smallest *p*-values for DILI prediction. Subsequently, we assessed feature importance using SHAP (Figure 2) [27], and the detailed descriptions of these features can be found in Supplementary Table S1.

### 3.2. Model Development and Performance Assessments

Table 1 presents a comparison of several machine learning methods in terms of performance measures evaluated by 100 repeats of 10-fold cross-validation. 

RF had the highest ACC of 0.631, followed by MLP (0.626) and SVM (0.617). RF also had the highest recall of 0.716, followed by KNN (0.664) and MLP (0.658). It achieved the highest F1 score value of 0.677, followed by MLP (0.660) and SVM (0.654). Meanwhile, RF had the lowest standard deviation in the recall and F1 score.

MLP had the highest MCC means (0.245), followed by RF and SVM (0.226 and 0.225, respectively). It also had the highest precision (0.673), followed by SVM (0.663) and NB (0.662). MLP recorded the second highest recall (0.658) and F1 score (0.660). 

Considering the metrics, RF consistently performs well in terms of ACC, precision, recall, MCC, and F1 score. It has the highest or near-highest mean values in most metrics. MLP has the highest MCC and precision and performs well in terms of recall. In summary, RF and MLP seem to be the best models based on the metrics provided. 

**Figure 3:**
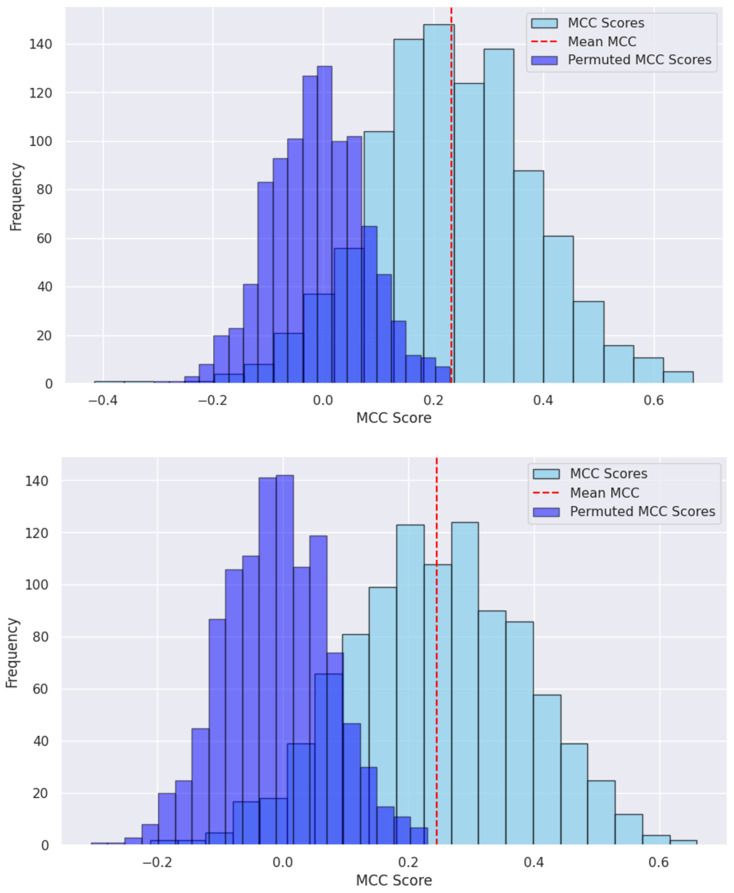
MCC scores from the permuted labels vs. the real labels by RF (**upper**) and MLP models (**down**). Both tests were statistically significant (*p*-value < 0.001).

We conducted a permutation test for the selected QSAR models based on RF and MLP. By randomly shuffling the labels, we generated a permuted label distribution. The *MCCs* from our RF and MLP models (Figure 3) were significantly higher than those obtained from the models with permuted labels (*p*-value < 0.0001). This suggests that the predictions from our models are not due to chance.

**Figure 4:**
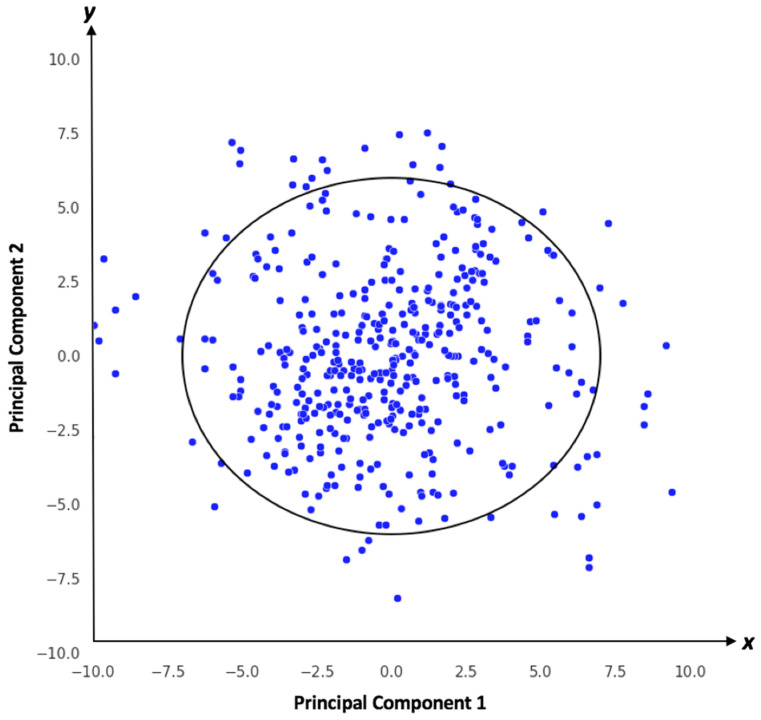
An applicability domain defines the specific conditions under which a predictive model or algorithm is expected to perform well. The blue dots are the training data points and elliptic circle indicates domain threshold.

We evaluated the RF model for the application domain. For threshold 0.6 (Figure 4), we observed the number of training data points inside the application domain to be 428 and the number of train data points outside the domain to be 32. Accordingly, the ACC within the domain is 0.64 and outside is 0.62.

### 3.3. Model Validation by Drug Candidates That Failed in Development

We tested the models using a set of 22 drug candidates that failed in drug development due to hepatotoxicity [15]. Both RF and MLP classifiers successfully predicted whether medications would result in “No-DILI-Concern” or “Most-DILI-Concern” with a success rate of 19/22 (90.9%) (Table 2). As a comparison, the rule-of-two model correctly predicted 13/22 compounds (59%), of which 8 compounds (*) were determined as ambiguous with either a high daily dose of >100 mg/day or a high logP value of >3 alone. We also applied the ToxSTAR online tool [28] for evaluating these drug candidates, which resulted in the correct prediction of 18/22 compounds (82%) for cholestasis and 16/22 compounds (73%) for hepatitis. 

## 4. Discussions

In this study, we endeavored to construct computer models for assessing DILI risk caused by medications, utilizing a substantial human dataset annotated by experts from LiverTox. To achieve this, an extensive exploration of various machine learning and deep learning techniques was undertaken to evaluate the efficacy of different modeling classifiers.

A 10-fold cross-validation procedure was employed to assess several selected machine learning algorithms, complemented by external testing. Our findings revealed that the RF and MLP models stood out as highly effective in predicting DILI. Specifically, RF outperformed other machine learning strategies, achieving an average prediction accuracy of 0.631 during cross-validation, while the MLP deep learning approach attained the best MCC of 0.245. The permutation tests revealed that the predictions from both models are better than by chance. 

QSAR models for DILI have been extensively studied in the literature. It is crucial to recognize that different endpoints in DILI prediction models can yield varying predictions due to differences in underlying mechanisms, data availability, and model complexities. In this study, we utilized a large dataset of drugs with quality endpoints related to DILI likelihood, as curated by the NIH’s LiverTox [11]. To the best of our knowledge, no other QSAR DILI model has been specifically based on the same endpoint. Another widely used dataset for developing QSAR models is DILIrank [29]. As detailed in Supplementary Table S2, various algorithms were employed to build these models, and they demonstrated comparable performance to ours, achieving accuracies in the range of 60–70% [8,30,31,32,33,34]. 

The top selected features by our models include Geary topological structure autocorrelation length-1 weighted by atomic van der Waals volumes (D455), Moran topological structure autocorrelation length-2 weighted by atomic masses (D480), the number of group Al-C(=X)-Al (D742), structure lopping centric group index (D253), and mean molecular topological order-9 charge index (D529). Interestingly, our top two ranking features have also been reported to contribute to QSAR models for predicting the inhibition of Cytochrome P450 enzymes, specifically CYP3A4, CYP2D6 [35] and CYP2C8 [36]. Notably, the inhibition of CYP enzymes is known to be associated with an increased risk of DILI in humans [37].

The RF model exhibited competitive F1 and AUC scores, demonstrating its capacity to successfully discriminate between positive and negative occurrences in the prediction process, achieving a harmonious balance between precision and recall. Of significant note, both models demonstrated their accuracy when externally validated against a set of 22 failed drug candidates with hepatotoxicity, although false negative rates need further evaluation. This underscores their potential in identifying compounds with hepatotoxic liabilities, mitigating risks to patients, and enhancing drug development procedures.

Both MLP and RF have unique advantages when developing QSAR models. RF offers robustness through its ensemble of decision trees, feature importance analysis, and noise resilience. On the other hand, MLPs excel at automatically learning from complex features and identifying non-linear connections between molecular descriptors and DILI. Without the need for additional ensemble methodologies, MLPs and RF can independently achieve competitive performance in DILI prediction by leveraging their respective strengths in feature learning and ensemble learning. These methods help identify important structural traits associated with DILI, providing a deeper understanding of the connections between chemical properties and liver damage.

However, there are also drawbacks to applying machine learning methods. One challenge is the availability of labeled data—finding high-quality, readily available data for training can be difficult. The size, quality, and representativeness of training datasets often vary, affecting the broad applicability of models. Another issue is class imbalance in datasets used for DILI prediction. For instance, when the proportion of liver injury cases is much lower than non-liver injury cases, models may perform poorly on the minority class and favor the dominant class. Extracting relevant traits or biomarkers from complex biological data can also be challenging. While machine learning algorithms can handle high-dimensional data, their interpretability may be limited, making it hard to understand the biological processes underlying DILI prediction. Additionally, complex models like deep learning neural networks (such as MLP) are prone to overfitting, especially when trained on small or noisy datasets. Overfitting occurs when a model memorizes the training data rather than capturing underlying patterns, leading to poor generalization performance on unseen data.

Developing a robust in silico model to predict the DILI risk in humans remains an unmet challenge in drug development. The urgency for accurate DILI prediction in pharmaceutical research and patient safety underscores the need for improved risk management and drug development practices. Machine learning approaches offer a promising solution to address these complex healthcare concerns. This study paves the way for future research into the application of machine learning in DILI prediction and its broader implications for medication safety and healthcare.

## Figures and Tables

**Table 1:** Performance matrices for different machine learning and deep learning models on DILI prediction (mean ± standardized deviation).

Model	Accuracy	Precision	Recall	*MCC*	*F*1
RF	0.631 ± 0.072	0.651 ± 0.092	0.716 ± 0.096	0.226 ± 0.145	0.677 ± 0.073
SVM	0.617 ± 0.071	0.663 ± 0.096	0.656 ± 0.099	0.225 ± 0.143	0.654 ± 0.079
DT	0.571 ± 0.077	0.623 ± 0.102	0.609 ± 0.102	0.132 ± 0.156	0.610 ± 0.084
KNN	0.600 ± 0.070	0.640 ± 0.095	0.664 ± 0.095	0.188 ± 0.146	0.648 ± 0.074
MLP	0.626 ± 0.068	0.673 ± 0.097	0.658 ± 0.096	0.245 ± 0.141	0.660 ± 0.077

**Table 2:** Model predictions for drug candidates terminated in drug development with hepatotoxicity findings.

Failed Drug Candidates	Rule-of-Two Model Prediction ^a^	RF/MLP Model Prediction
ADX-10059	Most-DILI-Concern	Most-DILI-Concern
Aplaviroc	Most-DILI-Concern	Most-DILI-Concern
CP-085958	Most-DILI-Concern	Most-DILI-Concern
CP-368296	Ambiguous *	Most-DILI-Concern
CP-422935	Most-DILI-Concern	Most-DILI-Concern
CP-456773	Most-DILI-Concern	Most-DILI-Concern
CP-457920	Ambiguous *	Most-DILI-Concern
CP-724714	Most-DILI-Concern	Most-DILI-Concern
Darbufelone	Ambiguous *	No-DILI-Concern
Falnidamol	Most-DILI-Concern	Most-DILI-Concern
Fialuridine	No-DILI-Concern	Most-DILI-Concern
Fiduxosin	Most-DILI-Concern	Most-DILI-Concern
LY-2409021	Ambiguous *	Most-DILI-Concern
MK-0893	Most-DILI-Concern	Most-DILI-Concern
Pafuramidine	Most-DILI-Concern	Most-DILI-Concern
Pralnacasan	Ambiguous *	No-DILI-Concern
Sitaxentan	Most-DILI-Concern	Most-DILI-Concern
Solithromycin	Most-DILI-Concern	Most-DILI-Concern
TAK-875	Ambiguous *	Most-DILI-Concern
Tasosartan	Ambiguous *	Most-DILI-Concern
Telcagepant	Most-DILI-Concern	Most-DILI-Concern
Zamifenacin	Ambiguous *	Most-DILI-Concern

^a^ The rule-of-two predictions were adopted from Leeson PD. (2018) [13]. * These compounds were determined as ambiguous in Leeson PD. (2018) [13] with either a high daily dose of >100 mg/day or a high logP value of >3 alone.

## Data Availability

The datasets used and/or analyzed during the current study are available from the corresponding author on reasonable request.

## Supplementary Materials

The following supporting information can be downloaded at: https://www.mdpi.com/article/10.3390/toxics12060385/s1, Table S1: Top 10 ranking features out of 168 lasso selected features with symbol and chemical definition; Table S2: The published QSAR models for predicting liver toxicity in humans using DILIrank dataset as the endpoint.

## References

[1] Weaver R.J., Blomme E.A., Chadwick A.E., Copple I.M., Gerets H.H.J., Goldring C.E., Guillouzo A., Hewitt P.G., Ingelman-Sundberg M., Jensen K.G. (2020). Managing the challenge of drug-induced liver injury: A roadmap for the development and deployment of preclinical predictive models. Nat. Rev. Drug Discov..

[2] Raschi E., Poluzzi E., Koci A., Salvo F., Pariente A., Biselli M., Moretti U., Moore N., De Ponti F. (2015). Liver injury with novel oral anticoagulants: Assessing post-marketing reports in the US Food and Drug Administration adverse event reporting system. Br. J. Clin. Pharmacol..

[3] Ashby K., Zhuang W., Gonzalez-Jimenez A., Alvarez-Alvarez I., Lucena M.I., Andrade R.J., Aithal G.P., Suzuki A., Chen M. (2021). Elevated bilirubin, alkaline phosphatase at onset, and drug metabolism are associated with prolonged recovery from DILI. J. Hepatol..

[4] George N., Chen M., Yuen N., Hunt C.M., Suzuki A. (2018). Interplay of gender, age and drug properties on reporting frequency of drug-induced liver injury. Regul. Toxicol. Pharmacol..

[5] Chen M., Bisgin H., Tong L., Hong H., Fang H., Borlak J., Tong W. (2014). Toward predictive models for drug-induced liver injury in humans: Are we there yet?. Biomark. Med..

[6] Dirven H., Vist G.E., Bandhakavi S., Mehta J., Fitch S.E., Pound P., Ram R., Kincaid B., Leenaars C.H.C., Chen M. (2021). Performance of preclinical models in predicting drug-induced liver injury in humans: A systematic review. Sci. Rep..

[7] Bassan A., Alves V.M., Amberg A., Anger L.T., Auerbach S., Beilke L., Bender A., Cronin M.T., Cross K.P., Hsieh J.-H. (2021). In silico approaches in organ toxicity hazard assessment: Current status and future needs in predicting liver toxicity. Comput. Toxicol..

[8] Chen M., Hong H., Fang H., Kelly R., Zhou G., Borlak J., Tong W. (2013). Quantitative Structure-Activity Relationship Models for Predicting Drug-Induced Liver Injury Based on FDA-Approved Drug Labeling Annotation and Using a Large Collection of Drugs. Toxicol. Sci..

[9] Mahalmani V., Sinha S., Prakash A., Medhi B. (2022). Translational research: Bridging the gap between preclinical and clinical research. Indian J. Pharmacol..

[10] Yu H.W. (2016). Bridging the translational gap: Collaborative drug development and dispelling the stigma of commercialization. Drug Discov. Today.

[11] Björnsson E.S., Hoofnagle J.H. (2016). Categorization of drugs implicated in causing liver injury: Critical assessment based on published case reports. Hepatology.

[12] (2012). Categorization of the Likelihood of Drug Induced Liver Injury. LiverTox: Clinical and Research Information on Drug-Induced Liver Injury.

[13] Leeson P.D. (2018). Impact of Physicochemical Properties on Dose and Hepatotoxicity of Oral Drugs. Chem. Res. Toxicol..

[14] Hong H., Xie Q., Ge W., Qian F., Fang H., Shi L., Su Z., Perkins R., Tong W. (2008). Mold^2^, Molecular Descriptors from 2D Structures for Chemoinformatics and Toxicoinformatics. J. Chem. Inf. Model..

[15] Fonti V., Belitser E. (2017). Feature selection using lasso. VU Amst. Res. Pap. Bus. Anal..

[16] Zhan X., Wang F., Gevaert O. (2022). Reliably Filter Drug-Induced Liver Injury Literature with Natural Language Processing and Conformal Prediction. IEEE J. Biomed. Health Inform..

[17] Hong H., Zhu J., Chen M., Gong P., Zhang C., Tong W. (2018). Quantitative structure–activity relationship models for predicting risk of drug-induced liver injury in humans. Drug-Induc. Liver Toxic..

[18] Aguirre-Plans J., Piñero J., Souza T., Callegaro G., Kunnen S.J., Sanz F., Fernandez-Fuentes N., Furlong L.I., Guney E., Oliva B. (2021). An ensemble learning approach for modeling the systems biology of drug-induced injury. Biol. Direct.

[19] Wainberg M., Alipanahi B., Frey B.J. (2016). Are random forests truly the best classifiers?. J. Mach. Learn. Res..

[20] Kawaguchi K., Lu H. Ordered sgd: A new stochastic optimization framework for empirical risk minimization. Proceedings of the International Conference on Artificial Intelligence and Statistics, PMLR.

[21] Shamir O., Zhang T. Stochastic gradient descent for non-smooth optimization: Convergence results and optimal averaging schemes. Proceedings of the International Conference on Machine Learning, PMLR.

[22] Chierici M., Francescatto M., Bussola N., Jurman G., Furlanello C. (2020). Predictability of drug-induced liver injury by machine learning. Biol. Direct..

[23] Dalianis H. (2018). Evaluation metrics and evaluation. Clinical Text Mining: Secondary Use of Electronic Patient Records.

[24] Hanser T., Barber C., Marchaland J.F., Werner S. (2016). Applicability domain: Towards a more formal definition. SAR QSAR Environ. Res..

[25] Tropsha A., Gramatica P., Gombar V.K. (2003). The Importance of Being Earnest: Validation is the Absolute Essential for Successful Application and Interpretation of QSPR Models. QSAR Comb. Sci..

[26] McLachlan G.J. (1999). Mahalanobis distance. Resonance.

[27] Wang H., Liang Q., Hancock J.T., Khoshgoftaar T.M. (2024). Feature selection strategies: A comparative analysis of SHAP-value and importance-based methods. J. Big Data.

[28] Shin H.K., Chun H.S., Lee S., Park S.M., Park D., Kang M.G., Hwang S., Oh K.H., Han H.Y., Kim W.K. (2022). ToxSTAR: Drug-induced liver injury prediction tool for the web environment. Bioinformatics.

[29] Chen M., Suzuki A., Thakkar S., Yu K., Hu C., Tong W. (2016). DILIrank: The largest reference drug list ranked by the risk for developing drug-induced liver injury in humans. Drug Discov. Today.

[30] Xu Y., Dai Z., Chen F., Gao S., Pei J., Lai L. (2015). Deep learning for drug-induced liver injury. J. Chem. Inf. Model..

[31] Li T., Tong W., Roberts R., Liu Z., Thakkar S. (2020). DeepDILI: Deep Learning-Powered Drug-Induced Liver Injury Prediction Using Model-Level Representation. Chem. Res. Toxicol..

[32] Wu L., Liu Z., Auerbach S., Huang R., Chen M., McEuen K., Xu J., Fang H., Tong W. (2017). Integrating Drug’s Mode of Action into Quantitative Structure–Activity Relationships for Improved Prediction of Drug-Induced Liver Injury. J. Chem. Inf. Model..

[33] Hong H., Thakkar S., Chen M., Tong W. (2017). Development of Decision Forest Models for Prediction of Drug-Induced Liver Injury in Humans Using A Large Set of FDA-approved Drugs. Sci. Rep..

[34] Zhang H., Ding L., Zou Y., Hu S.-Q., Huang H.-G., Kong W.-B., Zhang J. (2016). Predicting drug-induced liver injury in human with Naïve Bayes classifier approach. J. Comput. Mol. Des..

[35] McPhail B., Tie Y., Hong H., Pearce B.A., Schnackenberg L.K., Ge W., Valerio L.G., Fuscoe J.C., Tong W., Buzatu D.A. (2012). Modeling Chemical Interaction Profiles: I. Spectral Data-Activity Relationship and Structure-Activity Relationship Models for Inhibitors and Non-inhibitors of Cytochrome P450 CYP3A4 and CYP2D6 Isozymes. Molecules.

[36] Nembri S., Grisoni F., Consonni V., Todeschini R. (2016). In Silico Prediction of Cytochrome P450-Drug Interaction: QSARs for CYP3A4 and CYP2C9. Int. J. Mol. Sci..

[37] Yu K., Geng X., Chen M., Zhang J., Wang B., Ilic K., Tong W. (2014). High Daily Dose and Being a Substrate of Cytochrome P450 Enzymes Are Two Important Predictors of Drug-Induced Liver Injury. Drug Metab. Dispos..

