# Supplementary material for: Machine Learning to Predict Drug-Induced Liver Injury and Its Validation on Failed Drug Candidates in Development
Source: Toxics. 2024 May 24;12(6):385. doi: 10.3390/toxics12060385 (PMC11207878; doi:10.3390/toxics12060385)
Supplement: Supplementary file 1 [file toxics-12-00385-s001.zip › toxics-3001176-supplementary.pdf]

Supplemental Table S1: Top 10 ranking features out of 168 lasso selected features with symbol and chemical definition.

Symbol	Description
D455	Geary topological structure autocorrelation length-1 weighted by atomic van der Waals volumes
D480	Moran topological structure autocorrelation length-2 weighted by atomic masses
D742	number of group Al-C(=X)-Al
D253	structure lopping centric group index
D529	Mean molecular topological order-9 charge index
D471	number of group Ar-CH=X
D349	molecular topological path index of order 09
D745	number of group X-C(=X)-X
D759	number of group Al3-N
D675	number of group phenols

Supplemental Table S2. The published QSAR models for predicting liver toxicity in humans using DILIrank dataset as the endpoint.

Algorithm for modeling	Drugs for modeling	Overall performance	References
Decision forest	197 drugs for training and 190 drugs for test	68.9% accuracy for test	[8]
Multilayer Perceptron	475 for training and 198 drugs for test	70.3% accuracy in test	[30]
Naive Bayes classifier	420 drugs for training and 84 drugs for test	72% accuracy in test	[34]
Decision forest	721 drugs for training	72.9% accuracy in cross-validation	[33]
Decision forest	222 drugs for training and 111 for test	66.2% accuracy in test	[32]
Deep Neural Network, K-nearest, Decision forest, Support Vector Machine	753 for training and 249 drugs for test	65.8% balanced accuracy in test	[31]
